# Supplementary material for: Nerve growth factor receptor limits inflammation to promote remodeling and repair of osteoarthritic joints
Source: Nat Commun. 2024 Apr 15;15:3225. doi: 10.1038/s41467-024-47633-6 (PMC11018862; doi:10.1038/s41467-024-47633-6)
Supplement: Supplementary file 3 — Reporting Summary [file 41467_2024_47633_MOESM3_ESM.pdf]

nature portfolio

Corresponding author(s): Jian Huang

Last updated by author(s): Feb 29, 2024

Reporting Summary

Nature Portfolio wishes to improve the reproducibility of the work that we publish. This form provides structure for consistency and transparency in reporting. For further information on Nature Portfolio policies, see our Editorial Policies and the Editorial Policy Checklist.

Statistics

For all statistical analyses, confirm that the following items are present in the figure legend, table legend, main text, or Methods section.

n/a	Confirmed	
☐	☒	The exact sample size (*n*) for each experimental group/condition, given as a discrete number and unit of measurement
☐	☒	A statement on whether measurements were taken from distinct samples or whether the same sample was measured repeatedly
☐	☒	The statistical test(s) used AND whether they are one- or two-sided<br>*Only common tests should be described solely by name; describe more complex techniques in the Methods section.*
☒	☐	A description of all covariates tested
☒	☐	A description of any assumptions or corrections, such as tests of normality and adjustment for multiple comparisons
☐	☒	A full description of the statistical parameters including central tendency (e.g. means) or other basic estimates (e.g. regression coefficient) AND variation (e.g. standard deviation) or associated estimates of uncertainty (e.g. confidence intervals)
☐	☒	For null hypothesis testing, the test statistic (e.g. *F*, *t*, *r*) with confidence intervals, effect sizes, degrees of freedom and *P* value noted<br>*Give P values as exact values whenever suitable.*
☒	☐	For Bayesian analysis, information on the choice of priors and Markov chain Monte Carlo settings
☒	☐	For hierarchical and complex designs, identification of the appropriate level for tests and full reporting of outcomes
☒	☐	Estimates of effect sizes (e.g. Cohen's *d*, Pearson's *r*), indicating how they were calculated

Our web collection on statistics for biologists contains articles on many of the points above.

Software and code

Policy information about availability of computer code

Data collection	CellSens Imaging Software Version 4.1; Image Lab Software Version 3.0.1; ZEN microscopy software Version 3.7; CFX Manage Software Version 3.1
Data analysis	R Version 4.4.1; Seurat package (Version: 4.9.9.9060); CellChat Version 2.1.0; GraphPad Prism Version 6.0

For manuscripts utilizing custom algorithms or software that are central to the research but not yet described in published literature, software must be made available to editors and reviewers. We strongly encourage code deposition in a community repository (e.g. GitHub). See the Nature Portfolio guidelines for submitting code & software for further information.

Data

Policy information about availability of data

All manuscripts must include a data availability statement. This statement should provide the following information, where applicable:

- Accession codes, unique identifiers, or web links for publicly available datasets
- A description of any restrictions on data availability
- For clinical datasets or third party data, please ensure that the statement adheres to our policy

The data that support the findings of this study are available within the article, its supplementary Information files, and source data. The dataset analyzed in this study from Ref. 38 is available from the Gene Expression Omnibus (GEO) repository under the following accession number GSE143753.

Research involving human participants, their data, or biological material

Policy information about studies with human participants or human data. See also policy information about sex, gender (identity/presentation), and sexual orientation and race, ethnicity and racism.

Reporting on sex and gender	Human subjects were not recruited or involved in the study and human specimens used in the project were obtained from tissue repositories and de-identified prior to use. The samples were used when they became available. We used the cartilage samples based on the availability and whether they were osteoarthritic or not, thus we did not consider sex and gender in study design. All 5 control cartilage samples were from males and OA samples were from 3 males and 2 females.
Reporting on race, ethnicity, or other socially relevant groupings	Human subjects were not recruited or involved in the study and human specimens used in the project were obtained from tissue repositories and de-identified prior to use. The samples were used when they became available. We used the cartilage samples based on the availability and whether they were osteoarthritic or not, and we did not collect the information of race, ethnicity, or other socially relevant groupings from the tissue provider.
Population characteristics	Human subjects were not recruited or involved in the study and human specimens used in the project were obtained from tissue repositories and de-identified prior to use. The samples were used when they became available. The average age of the donors of the control cartilage samples were 54.2 years and that of OA samples were 71.4 years. We used the cartilage samples based on the availability and whether they were osteoarthritic or not, thus we could not consider age in study design.
Recruitment	Human subjects were not recruited or involved in the study and human specimens used in the project were obtained from tissue repositories and de-identified prior to use. The samples were used when they became available.
Ethics oversight	Human subjects were not recruited or involved in the study and human specimens used in the project were obtained from tissue repositories and de-identified prior to use. The study has been approved by the IRB of Rush University Medical Center.

Note that full information on the approval of the study protocol must also be provided in the manuscript.

Field-specific reporting

Please select the one below that is the best fit for your research. If you are not sure, read the appropriate sections before making your selection.

☒ Life sciences ☐ Behavioural & social sciences ☐ Ecological, evolutionary & environmental sciences

For a reference copy of the document with all sections, see nature.com/documents/nr-reporting-summary-flat.pdf

Life sciences study design

All studies must disclose on these points even when the disclosure is negative.

Sample size	Sample size is described in the figure legends. No calculations were performed to determine the sample size. Sample sizes were determined based on previous studies, as cited in the Methods of the manuscript, such as Ref. 19 and 31.
Data exclusions	No data was excluded.
Replication	All experiments were performed at least 3 times unless otherwise stated in the figures or figure legends. The number of biological replicates are described in the manuscript, and the results are reproducible across independent experiments.
Randomization	Cells and animals of each genotype were randomly allocated to different study group.
Blinding	The tests were performed in a blind manner that the investigator is not aware of the identification of animals as well as the study groups, except those tests that were not practical for blinding like Western blot and RT-qPCR.

Reporting for specific materials, systems and methods

We require information from authors about some types of materials, experimental systems and methods used in many studies. Here, indicate whether each material, system or method listed is relevant to your study. If you are not sure if a list item applies to your research, read the appropriate section before selecting a response.

Materials & experimental systems

n/a	Involved in the study
☐	☒ Antibodies
☐	☒ Eukaryotic cell lines
☒	☐ Palaeontology and archaeology
☐	☒ Animals and other organisms
☒	☐ Clinical data
☒	☐ Dual use research of concern
☒	☐ Plants

Methods

n/a	Involved in the study
☒	☐ ChIP-seq
☒	☐ Flow cytometry
☒	☐ MRI-based neuroimaging

Antibodies

Antibodies used

anti-NGFR antibody (Cell Signaling, catalog # 8238S), anti-RUNX2 antibody (MBL, catalog # D130-3), anti-NGF antibody (abcam, catalog # ab6199), anti-p-SMAD1 (Cell Signaling, catalog # 9516S), anti-SMAD1 (abcam, catalog # ab63356); anti-p65 antibody (Cell Signaling, catalog #8242), anti-RANKL (Novus Biologicals, Clone 12A668), anti-osteoprotegerin (Novus Biologicals, Clone 98A1071), anti-βIII-tubulin antibody (R&D Systems, catalog # MAB1195); anti-phospho-NF-κB p65 (Ser536), Cell Signaling, catalog #3033; anti-phospho-IκBα (Ser32), Cell Signaling, catalog # 2859; anti-IκBα, Novus Biologicals, catalog # NB100-56507; anti- Phospho-IKKα (Ser176)/IKKβ (Ser177), Cell Signaling, catalog #2078; anti-IKKβ, Cell Signaling, catalog #2678; anti-β-actin, Sigma-Aldrich, catalog # A5441; TrkA Antibody, Cell Signaling, catalog #2505; Trk (pan) (A7H6R) Rabbit mAb, Cell Signaling, catalog #92991

Validation

The antibodies were commercially available. In Western blots, we used molecular wright of the detected band and negative control (knockout or cytokine-untreated) or positive control (overexpression or treated) as a validation strategy, as in Fig. 2c, 2d, 5b, 5c, 8a, 8b, 8c, 8d, 8e. In IHC, we used the cell types expressing the antigens as well as negative control (knockout or no DMM surgery) or positive control (diseased tissues) as a validation strategy, as in Fig. 1e, 4b, 5g, 5h, 5j, 7a, 7c. Moreover, these antibodies are all well-cited. For the citation information and the vendor's validation information, please see the following:
https://www.cellsignal.com/products/primary-antibodies/p75ntr-d4b3-xp-rabbit-mab/8238
https://www.mblintl.com/products/d130-3/
https://www.abcam.com/products/primary-antibodies/ngf-antibody-bsa-and-azide-free-ab6199.html
https://www.cellsignal.com/products/primary-antibodies/phospho-smad1-5-ser463-465-41d10-rabbit-mab/9516
https://www.abcam.com/products/primary-antibodies/smad1-antibody-ab63356.html
https://www.cellsignal.com/products/primary-antibodies/nf-kb-p65-d14e12-xp-rabbit-mab/8242
https://www.novusbio.com/products/trance-tnfsf11-rank-l-antibody-12a668_nb100-56512
https://www.novusbio.com/products/osteoprotegerin-tnfrsf11b-antibody-98a1071_nb100-56505
https://www.rndsystems.com/products/neuron-specific-beta-iii-tubulin-antibody-tuj-1_mab1195
https://www.cellsignal.com/products/primary-antibodies/phospho-nf-kb-p65-ser536-93h1-rabbit-mab/3033
https://www.cellsignal.com/products/primary-antibodies/phospho-ikba-ser32-14d4-rabbit-mab/2859
https://www.novusbio.com/products/ikb-alpha-antibody-6a920_nb100-56507
https://www.cellsignal.com/products/primary-antibodies/phospho-ikka-ser176-ikkb-ser177-c84e11-rabbit-mab/2078
https://www.cellsignal.com/products/primary-antibodies/ikkb-l570-antibody/2678
https://www.sigmaaldrich.com/US/en/product/sigma/a5441
https://www.cellsignal.com/products/primary-antibodies/trka-antibody/2505
https://www.cellsignal.com/products/primary-antibodies/trk-pan-a7h6r-rabbit-mab/92991

Eukaryotic cell lines

Policy information about cell lines and Sex and Gender in Research

Cell line source(s)

Cell lines such as C3H10T1/2, ST2, C2C12, ATDC5, RCS, hFOB1.19, Saos-2, and human mesenchymal stem cells are available and maintained in our lab. Mouse articular chondrocytes and limb bud cells were isolated from the mouse newborns or embryos, of which the sex information was not obtained due to their small sizes.

Authentication

We routinely culture the cell lines and validate the cells through morphology, differentiation, and molecular examinations.

Mycoplasma contamination

Not tested for mycoplasma contamination.

Commonly misidentified lines
(See ICLAC register)

None.

Animals and other research organisms

Policy information about studies involving animals; ARRIVE guidelines recommended for reporting animal research, and Sex and Gender in Research

Laboratory animals

The animal protocol of this study has been approved by the Institutional Animal Care and Use Committee (IACUC) of the Rush University Medical Center and all experimental methods and procedures were carried out in accordance with the approved

guidelines. Mice were maintained in the Rush University Medical Center Comparative Research Center. Animals were housed in the Micro-Isolator® system at 22°C temperature, 45% humidity, and 12-hour light/dark cycle, and given food (2018 Teklad Global Rodent Diet) and water ad libitum. The Ngfr conditional knockout mice used in this study had heterozygous (a single copy) Acan-CreER and homozygous floxed Ngfr (JAX Strain # 031162). Their Cre-negative littermates were used as the control mice. Sex-matched control and NGFR KO mice were used. We performed the DMM surgery on the right knee of the 3-month-old mice to induce OA. Under anesthesia induced and maintained by isoflurane, the hind limbs were shaved and prepared for aseptic surgery. The right knee joint was exposed following a medial capsular incision and gentle lateral displacement of the extensor mechanism without transection of the patellar ligament. The medial meniscotibial ligament was dissected. After replacement of the extensor and irrigation with saline to remove tissue debris, the medial capsular incision and the skin incision were closed. We monitored for general signs of stress and pain of the mice and found that the DMM surgery was well tolerated in the mice. Two strategies of tamoxifen injection (1 mg/10g body weight, i.p. injection) were used respectively: 5-day injections when the mice are 15 days old; or weekly injections starting from 10 days after DMM until sample harvest. Both the control mice and the NGFR KO mice received tamoxifen injection. The DMM surgery induced post-traumatic osteoarthritis in both females and males, and we did not observe significant differences regarding pathological changes in osteoarthritic joints between female and male mice after they were subjected to the DMM surgery. Thus, animal sex was not analyzed as a variable in this work. We also performed the DMM surgery on 6 male C57BL/6J mice to induce OA, which aimed to study the phenotypic and molecular changes during OA pathogenesis. We performed the sham operation on 6 male C57BL/6J mice by opening and exposing the structures of the knee and then closing the skin incision without manipulating joint tissues. Acan-CreER mice were crossed to Ai9 (Rosa-CAG-LSL-tdTomato-WPRE, Jackson Strain # 007909) to generate the Acan-CreER reporter mice, namely Ai9;Acan-CreER. Weekly injection of tamoxifen started from 10 days after DMM on 3-month-old male Ai9;Acan-CreER or Ai9 male mice (n = 3), until the samples were collected two months later. The mice were euthanized under carbon dioxide.

Wild animals	This study did not involve wild animals.
Reporting on sex	We used male mice in the studies on the pathogenic course of OA (Figure 1) and lineage tracing (Figure 4a). We used both female and male mice in the study on NGFR loss-of-function, in which the control and NGFR KO mice were sex-matched. The surgery of destabilization of the medial meniscus is an established model in inducing post-traumatic osteoarthritis in both females and males, and we did not observe significant differences regarding pathological changes in osteoarthritic joints between female and male mice after they were subjected to the surgery of destabilization of the medial meniscus. Thus, animal sex was not analyzed as a variable in this work.
Field-collected samples	This study did not involve samples collected from the field.
Ethics oversight	The animal protocol of this study has been approved by the Institutional Animal Care and Use Committee (IACUC) of the Rush University Medical Center and all experimental methods and procedures were carried out in accordance with the approved guidelines.

Note that full information on the approval of the study protocol must also be provided in the manuscript.

Plants

Seed stocks	This study did not involve plants.
Novel plant genotypes	This study did not involve plants.
Authentication	This study did not involve plants.
